# Impact of digital and conventional rehabilitation aftercare on physical and mental health in orthopedic patients in Germany

**DOI:** 10.3389/fpubh.2024.1344063

**Published:** 2024-07-05

**Authors:** Detlef Schmidt, Julian Fritsch, Katharina Feil, Susanne Weyland, Lena-Marie Rittmann, Darko Jekauc

**Affiliations:** ^1^DRV Knappschaft-Bahn-See, Bochum, Germany; ^2^Karlsruhe Institute of Technology, Karlsruhe, Germany

**Keywords:** traditional aftercare, digital aftercare, health, digital rehabilitation, intervention, rehabilitation

## Abstract

The integration of digital interventions in health rehabilitation offers promising opportunities to improve patient outcomes. However, empirical studies comparing the effectiveness of digital and traditional rehabilitation interventions remain scarce. This study was designed to evaluate the impact of a digital aftercare program, compared to traditional aftercare and a control group, on both psychological and physical health outcomes in individuals undergoing orthopedic rehabilitation. Additionally, the study also aimed to examine the moderating effects of age and gender. The study employed a partially controlled trial design, engaging a cohort of 805 orthopedic patients, divided into: digIRENA (*n* = 323, digital aftercare), IRENA (*n* = 252, traditional aftercare), and a control group (*n* = 230, without organized aftercare). Measurements took place at four different time points: baseline (start of the rehabilitation program), T1 (13 weeks after the start of rehabilitation, marking the midpoint of aftercare), T2 (26 weeks, marking the end of aftercare), and T3 (43 weeks, to assess the sustainability of aftercare effects). The SF-12 Health Survey was the primary data collection instrument for measuring trends in physical and mental health outcomes over these intervals using repeated measures ANOVA. The results show that rehabilitants in the digIRENA group participated for a longer period of time than rehabilitants in the IRENA group, while the two groups did not differ in terms of motivation at T0 and organized physical activity outside of aftercare at T3. A significant improvement in physical health outcomes was observed in all groups across time, with digIRENA participants showing the greatest improvement. For mental health, all groups showed initial improvements, with the digIRENA group showing the most pronounced increase at T2. Overall, there was a decline in the effects achieved 4 months after the end of aftercare. When age and gender were included as covariates, the time effect for mental health disappeared, showing a significant time * gender interaction due to significantly lower baseline scores of women compared to men. The results of the study show that digital interventions, in particular the digIRENA program, contribute to improving health rehabilitation outcomes. The digIRENA program and similar digital health interventions may offer potential for improving health rehabilitation aftercare.

## 1 Introduction

Within the healthcare landscape, the German Pension Insurance (Deutsche Rentenversicherung) has systematically operationalized an array of rehabilitative interventions, both inpatient and outpatient, governed by the axioms of “rehabilitation before pension” and “rehabilitation before care” (1). Central to this rehabilitative framework is the objective of restoring individuals’ functional capacities and enhancing occupational performances after health-related setbacks (2). A secondary aim is the promotion of sustainable health behaviors and attenuation of detrimental lifestyle choices, thereby mitigating risks of disease relapse and overall health decline (3). Concurrently, demographic shifts indicate a diminishing working-age population, which poses dual challenges to the pension system. According to projections from the Federal Statistical Office (4), the proportion of working-age individuals (20–66 years) is anticipated to recede from 62 to 54% by 2060, while the population aged 67 and above is projected to rise from 20 to 28%. These trends caused an investment of €7.11 billion in rehabilitative interventions by the German Pension Insurance in 2018, highlighting the importance of rehabilitation for labor market sustainability (5).

The German healthcare system is characterized by its universal coverage, providing comprehensive health services to all citizens through a mix of statutory health insurance and private health insurance schemes. Central to this system’s approach to rehabilitation is the German Pension Insurance, which plays a pivotal role in operationalizing rehabilitative interventions aimed at facilitating individuals’ return to work and daily life activities. These interventions, governed by the principles of “rehabilitation before pension” and “rehabilitation before care,” emphasize the system’s preventive and restorative focus (6). Although initial rehabilitative interventions have demonstrated efficacy, longitudinal studies suggest a significant decline in their beneficial effects over time (7–9). This attenuation can be attributed to several factors, including the fading of initial motivation (10), challenges in maintaining new health behaviors in the absence of structured support (11), and the complex interplay of chronic conditions and lifestyle factors (12). Moreover, the effectiveness of rehabilitation is not only influenced by the content and delivery of the intervention but also by patients’ motivation and engagement, which are critical for sustaining long-term health benefits (13).

In response to this decline, the German Pension Insurance implemented the Intensified Rehabilitation Aftercare (IRENA) program. This program utilizes a multimodal approach, combining therapeutic methods from various disciplines to address an extensive range of conditions such as musculoskeletal, metabolic, cardiovascular, neurological, and psychological disorders (14). Accredited therapists within the IRENA program are required to have a minimum of 3 years of professional practice, ensuring that patients receive high-quality care from experienced practitioners. Structurally, the IRENA program incorporates strategies for occupational reintegration while emphasizing long-term health-promoting behaviors. The holistic nature of the IRENA program is designed to not only facilitate physical recovery but also to address the psychological well-being of patients, thereby underscoring the intrinsic link between physical and mental health in rehabilitation contexts. Physical rehabilitation is crucial for restoring function, improving mobility, and alleviating pain, which directly impacts an individual’s quality of life and mental health by enhancing self-efficacy, reducing stress, and mitigating the risk of depression often associated with physical ailments (15). Empirical data underscores the growing use of IRENA services, as reflected in the increasing number of approved aftercare interventions (5).

However, despite its comprehensive endeavors, the IRENA program has notable limitations in terms of accessibility and adaptability to individual needs (15). Empirical evidence suggests that both inpatient and outpatient paradigms lack sufficient spatiotemporal flexibility (16). Barriers such as occupational obligations, geographical remoteness, rigid schedules, and family responsibilities may impede access to the program. These challenges are exacerbated in rural areas where accessing aftercare facilities within a reasonable time becomes problematic. In addition, the rigid schedule of the IRENA program often conflicts with work commitments in areas such as hospitality and leisure, as well as family responsibilities including caregiving and childcare. This lack of flexibility can hinder the overall effectiveness of the rehabilitation process, potentially affecting patients’ motivation to engage and adhere to the aftercare program.

The incorporation of telerehabilitation technologies represents a viable strategy to enhance accessibility and offer a more person-centered approach by leveraging digital technologies. Using telecommunication technologies, telerehabilitation creates a more flexible healthcare delivery model, substantially reducing geographical and conflicting activities – factors particularly salient in rural areas and among populations with irregular working hours (17). This innovation could not only facilitate access to rehabilitation services, but also meet the motivational needs of patients by providing personalized and engaging digital content (18). However, research into telerehabilitation also highlights significant challenges, including technological accessibility issues, the need for digital literacy among participants, potential reductions in the personalization of care due to the absence of physical presence, and concerns regarding the privacy and security of health data (19, 20).

Meta-analytic evidence supports the equivalence of therapeutic outcomes between telerehabilitation and traditional face-to-face modalities for motor recovery and recovery from total knee arthroplasty (21, 22). By adopting telerehabilitation technologies into the rehabilitation aftercare, one can overcome the inflexible parameters inherent to traditional programs such as IRENA, thereby facilitating a more individualized, adaptive intervention paradigm. Consequently, the operationalization of telerehabilitation programs could lead to considerable enhancements in the accessibility and adaptability of rehabilitation aftercare programs, contributing to the optimization of patient outcomes and the overall efficiency of the healthcare infrastructure.

When evaluating the effectiveness of telerehabilitation aftercare interventions, it is necessary to acknowledge the heterogeneity in implementation modalities, ranging from telephonic support to web-based platforms offering comprehensive therapeutic modules. A meta-analysis focusing on web-based applications for breast cancer patients found generally positive effects on specific indicators such as therapy-related menopausal symptoms and sleep function (23). However, these results were inconsistent with respect to other indicators such as health-related quality of life and cognitive functions. Studies conducted within Germany, such as the one by Ebert et al. (24), have also reported positive results, demonstrating that patients who participated in web-based aftercare programs in addition to conventional approaches such as IRENA were more effective in stabilizing the treatment outcomes over a 12-month period. However, there remains a research gap, particularly concerning a detailed exploration of telerehabilitation’s effectiveness in aftercare settings. This includes aspects often overlooked in previous studies, such as comprehensive comparisons with control groups and a focused examination of both psychological and physical health outcomes.

The primary objective of the present study was to compare the effectiveness of the digital rehabilitation aftercare with traditional IRENA and a control group on physical and mental health outcomes among patients undergoing orthopedic rehabilitation. Additionally, the study aimed to examine the moderating effects of age and gender, as these variables have been shown to be predictors of physical and mental health (25) and as possible moderating effects of age on intervention effectiveness have been demonstrated (26).

## 2 Methods

### 2.1 Study design

The protocol for this study design has been published (27). A semi-randomized, longitudinal study design was used including three different study groups: traditional IRENA, digIRENA (digital IRENA), and a control group. All participants met the legal criteria for post-rehabilitation IRENA eligibility. The allocation of participants to the groups took place during their initial consultation with the responsible doctor at a rehabilitation clinic. Those rejecting IRENA participation were randomly allocated to either the digIRENA or control group. Sealed envelopes were used for the randomization procedure and the participants knew their group only after opening the envelopes. These envelopes were evenly distributed across the clinics to achieve a balanced 1:1 ratio between the digIRENA and control groups. It is important to note that, due to the transparent nature of the aftercare interventions, neither participants nor staff could be blinded to group allocation. Additionally, it was observed that some participants opted not to participate in the study upon learning they had been assigned to the control group rather than the digIRENA group, indicating a preference for the digital rehabilitation aftercare option. To increase the motivation to participate in the study, the participants that completed all four measurements received a 50 Euro Amazon voucher. Participants were initially recruited from three multiple rehabilitation clinics belonging to DRV Knappschaft-Bahn-See and the plan was to recruit from January 2020 until October 2020. However, recruitment was interrupted due to COVID-19 and also after the interruption there was a reduced admission in the rehabilitation clinics. For this reason, in August 2020 two more rehabilitation clinics joined and a one-year recruitment extension was authorized until October 2021. Because the number of sufficient participants was reached, the recruitment ended in August 2021. Although the initial plan was to recruit 1,150 participants at baseline (27), the dropout was less than expected and therefore the recruitment stopped when 1,060 participants were recruited.

The study comprised four measurement time points: pre-aftercare (T0), at 13 weeks post-initiation of aftercare (T1), at 26 weeks or the assumed end of aftercare (T2), and a 17-week post-aftercare follow-up (T3). These measurement intervals were strategically selected to capture the midpoint (T1), endpoint (T2), and a four-month follow-up (T3). It is critical to acknowledge that this schema represents an idealized timeline as deviations occurred due to varying starting time points and inconsistent adherence to the aftercare programs. Data collection included in-clinic questionnaires at T0 and subsequent mail or email sending of questionnaires at T1, T2, and T3, accompanied by a two-week non-response follow-up reminder. The study has been formally registered with the German Register of Clinical Studies under the identifier DRKS00022467. Ethical approval and data protection compliance have been obtained from the Ethics Committee and the Data Protection Officer at the Karlsruhe Institute of Technology.

### 2.2 Sample

Power calculations indicated the necessity for 573 participants to assess telerehabilitation aftercare effectiveness, assuming a small effect size (Cohen’s *f* = 0.07) due to limited robust empirical studies in this area (22). With an alpha error set at 0.05 and a desired test power of 0.80, this sample size is required when using analysis of variance with repeated measurement. Anticipating a dropout rate of about 30% per measurement point, the study initially aimed to enroll approximately 1,150 patients. Finally, because drop-out was lower than expected, 1,060 orthopedic rehabilitation patients were recruited at the baseline measurement (T0). In our study, we specifically targeted patients undergoing orthopedic rehabilitation, reflecting our primary aim to assess the effectiveness of digital and traditional aftercare within a cohort primarily affected by musculoskeletal disorders. Participants were recruited from five distinct rehabilitation clinics, ensuring a diverse sample representative of individuals undergoing orthopedic treatment. During their rehabilitation stay, the study’s objectives and procedures were outlined to potential participants in a medical consultation, where study information and consent forms were provided. Those who agreed to participate completed the questionnaire either during their hospital stay or directly after discharge. A key inclusion criterion was a sufficient command of the German language, ensuring that all participants could complete the questionnaire independently without requiring external assistance. Attrition occurred over the course of the study, with four participants withdrawing consent for data utilization (two from digIRENA, one from traditional IRENA, and one from the control group). Thus, at T0 the sample consisted of 405 individuals in the digIRENA intervention cohort (145 females, representing 35.8%), 352 in the traditional IRENA cohort (120 females, 34.1%), and 299 in the control cohort (79 females, 26.4%). With relevance for the analysis of the present study, there were 805 participants who filled out the questionnaires at all four measurement occasions. From these, 323 were in the digIRENA group, 252 in the IRENA group, and 230 in the control group.

### 2.3 Measures

#### 2.3.1 Health

Health-related variables were assessed using the SF-12 Health Status Questionnaire, an abbreviated version of the SF-36 (28). This instrument comprises two subscales that delineate physical and mental health dimensions. The SF-12 has been empirically validated as a robust tool for measuring subjective health states, independent of an individual’s current health status (28). The instrument employs a diverse array of response formats, including Likert scales as well as binary options. In calculating the SF-12 scores, we followed the original methodology proposed by Ware et al. (29). This decision was made to align with international research standards, facilitating comparability across studies. However, it is crucial to acknowledge that alternative scoring methods exist, such as those proposed by Wirtz et al. (30, 31). In relation to reliability metrics, existing literature reveals an internal consistency of Cronbach’s alpha = 0.83 for the Physical Health subscale and of 0.87 for the Mental Health subscale (28). With regard to content validity, the phrasing of the questionnaire items has been adjudicated as both comprehensible across diverse samples (32).

#### 2.3.2 Participation in aftercare

The participants of both the IRENA and digIRENA groups were asked if they had commenced their aftercare sessions since discharge from the respective clinic. For those who had already begun their aftercare, further inquiries were made regarding the number of weeks they participated in at least one aftercare session (i.e., the frequency of attendance) and the average duration spent in minutes per week attending these sessions (i.e., the duration of training). From these responses, the overall engagement in aftercare activities (in minutes) was calculated and compared between the two aftercare groups. For the purposes of this study, only the data at time t3 were analyzed, as this contained information on the extent of participation over the entire aftercare period.

#### 2.3.3 Participation in other structured physical activities

To comprehensively assess physical activity beyond the scope of rehabilitation aftercare, we incorporated three specific survey items targeting all participant groups. The first item queried participants on their involvement in any structured physical activities external to the designated aftercare programs. The subsequent item detailed the type of physical activities engaged in, while the third item captured the average weekly duration of these activities. For the purpose of this study, the first and third items were utilized at the second time point (t2) to estimate the overall duration of structured physical activity throughout the rehabilitation aftercare period. Data on the average duration of such activities, reported in minutes per week, were then incorporated into our analyses to account for additional physical engagements outside the primary aftercare interventions.

#### 2.3.4 Motivation

Motivation was assessed using the German translation of the Behavioral Regulation in Exercise Questionnaire-2 (BREQ-2) (33). This questionnaire consists of 19 items with a 5-point response format, designed to measure five different types of motivational regulation: (1) intrinsic motivation, (2) identified regulation, (3) introjected regulation, (4) external regulation, and (5) amotivation. In addition to these distinct motivational forms, the Relative Autonomy Index (RAI) can be calculated, which represents the degree of self-determination an individual exhibits toward a behavior (33). The RAI was calculated by multiplying the scale value of intrinsic motivation by three, identified motivation by two, introjected regulation by negative one, external motivation by negative two, and amotivation by negative three, then summing all five scale values. Regarding reliability, the internal consistency (Cronbach’s α) for the amotivation scale was 0.60, external regulation was 0.77, introjected regulation was 0.77, identified regulation was 0.83, and intrinsic regulation was 0.88 (34). In terms of validity, it has been demonstrated that the subscales correlate with each other in a theoretically consistent manner, and higher self-determined motivations are associated with a greater likelihood of engaging in health behaviors (34). This questionnaire was applied exclusively to the digIRENA and traditional IRENA groups to measure their motivation regarding participation in aftercare sessions. In order to determine the initial motivation of the participants, we only analyzed the information at t0 for the purposes of this study.

### 2.4 Intervention

All participants in the study were offered IRENA intervention, and those subjects who declined to participate in IRENA were randomly assigned to either the digIRENA group or the control group. Two intervention programs were implemented in this study: Traditional IRENA and digIRENA. Participants in both intervention programs were under the continuous supervision of accredited therapists. In the case of adverse outcomes, participants were referred for additional treatment. Additionally, a control cohort was established, without a prescribed aftercare intervention.

#### 2.4.1 Traditional IRENA

Every rehabilitant in Germany has a right to rehabilitation aftercare such as IRENA following rehabilitation. In general, IRENA courses are offered in health centers or clinics spread throughout the country and rehabilitants have the opportunity to register for these courses. The traditional IRENA program permits a maximum of 24 therapeutic sessions with a duration of 90–120 min each. The IRENA program covers treatments from at least two therapeutic fields or chapters of the Classification of Therapeutic Services in Medical Rehabilitation (KTL; Klassifikation thereapeutischer Leistungen in der medizinischen Rehabilitation), making it interdisciplinary and involving multiple professional groups. It serves insured individuals who need exercise for functional limitations and also require lifestyle and behavior stabilization through structured education. This approach combines therapeutic and psychoeducative services, involving both movement therapists and psychologists.

For musculoskeletal disorders, the IRENA concept is tailored to those rehabilitants who require a combination of treatment elements from different therapeutic directions and specific care, monitoring, and therapy in a specialized facility (6). The therapeutic activities within IRENA for musculoskeletal conditions might include, but are not limited to: (i) Group physiotherapeutic treatments focusing on musculoskeletal diseases, (ii) Water-based physiotherapeutic treatments for musculoskeletal conditions, (iii) Psychologically oriented group work targeting specific disorders, and (iv) Structured education on managing non-inflammatory diseases of the musculoskeletal system or chronic pain. This comprehensive approach aims at not just addressing the physical aspects of musculoskeletal disorders but also at managing the psychological impacts, thereby fostering a holistic recovery process. The emphasis is on enhancing functionality, reducing pain, and improving the overall quality of life while also addressing the psychological aspects such as coping strategies for pain management and fostering a proactive approach toward maintaining health (6).

#### 2.4.2 digIRENA

The Digital Intensified Rehabilitation Aftercare (digIRENA) program represents an innovativeadaptation of the established IRENA framework, incorporating digital technologies to improve the reach, customization, and participant engagement in post-rehabilitative care. Aimed at overcoming the barriers associated with traditional aftercare models, digIRENA introduces a tele-rehabilitation approach that offers flexibility, accessibility, and a focus on the patient’s experience. This is particularly beneficial for individuals hindered by geographical distance, physical limitations, or scheduling conflicts.

The core of digIRENA is the use of the Caspar app,^1^ which provides 24 flexible telerehabilitation sessions of up to 90 min each. The integration of Caspar into the patient treatment begins during their inpatient treatment, preparing them for a smooth transition to self-directed training under remote therapeutic supervision once discharged. The service suite of digIRENA is designed to meet the rehabilitation needs of patients with musculoskeletal and other conditions, offering digital therapeutic modules that encompass virtual physiotherapy, educational content on lifestyle and disease management, and platforms for psychological support, all accessible from any location.

The transition from inpatient care to digIRENA’s telerehabilitation is facilitated by Caspar’s comprehensive patient information transfer protocol, ensuring continuity of care. The telerehabilitation sessions are structured, featuring a combination of visual, auditory, and animated guidance, and are designed to encourage ongoing dialogue between the patient and the therapist. This interactive setup allows for the personalized adaptation of the rehabilitation plan based on direct feedback. The Caspar platform provides a robust system for tracking and documenting patient engagement and progress, including a feature for patients to record and share videos of their exercise performance. This input is centrally monitored through a “therapist dashboard,” enabling therapists to make immediate adjustments to the rehabilitation plan as needed.

#### 2.4.3 Control group

Participants in the control group were initially offered the opportunity to participate in the traditional IRENA program, but chose not to do so. Due to the random draw, they were not assigned to the digIRENA intervention and therefore did not receive any structured aftercare. However, this group retained the option to participate in physical activity programs, including participation in community-based rehabilitation or general exercise programs.

### 2.5 Statistical analyses

The acquired dataset underwent preliminary data processing prior to analysis. In a first step, missing data were analyzed separately for each measurement time point using Little’s MCAR test to determine the extent to which systematic missingness was present (35). Missing values at the item level were imputed utilizing an Expectation–Maximization algorithm (36), an iterative procedure consisting of expectation and maximization steps that converge upon a local maximum likelihood estimate (37). Post-imputation, values were rounded to the nearest integer. In cases where fewer than 50% of the required values were available for a given scale score, missing value imputation was precluded, and the corresponding sum score was reported as ‘missing,’ thereby excluding the data from subsequent analyses. The differences between the three groups in terms of dropout rate between t0 and t3 were analyzed using the chi-squared goodness of fit test. One participant who self-identified as “diverse” in gender was excluded from analyses using age and gender as covariates.

An intention-to-treat analysis was employed, encompassing all individuals who consented to participate in the study and for whom data were available. Descriptive statistics were presented with regard to the distribution of gender, age, nationality, employment status, participation in aftercare, and motivation. To examine the differences between the digIRENA group and the IRENA group in terms of motivation and attendance, t-test and Welch test were used. Thereafter, repeated measures analyses of variance (ANOVA) were conducted to interrogate longitudinal trends in dependent variables, specifically subjective physical, and subjective mental health metrics as assessed by the SF-12 questionnaire (38). ANOVA was used across all four measurement occasions to assess the effects of the aftercare intervention and its durability. The sphericity assumption was tested using the Mauchly test and, in case of violation, the Greenhouse–Geisser adjustment was used to correct for violations of sphericity. To accommodate the challenges posed by unbalanced sample sizes in our ANOVA analysis, we employed both Type II and Type III ANOVA, ensuring the robustness of our findings across the digIRENA, IRENA, and control groups. The similarity of results between these analyses suggests that our conclusions are reliable and unaffected by the choice of analytical method. Initial group allocation biases based on age and gender variables were examined through logistic regression. In a separate ANOVA, the study examined the interaction of time, group, and age, based on the study by Schmidt et al. (39), where such an interaction suggested that younger participants benefited more from digital prevention programs, while older participants benefited more from traditional approaches. For this analysis, median split was performed for the variable age. For all three groups, the mean curves over the four measurement time points were presented graphically, with the 95% confidence interval for each mean value. In case an interaction was significant, additional figures were presented. All analyses were performed at the 5% significance level using SPSS version 28.

## 3 Results

### 3.1 Missing data and descriptive statistics

Analysis of missing values for the SF-12 at the first measurement time point revealed that there were 51 missing values (0.40%) across 33 (3.13%) of all 1,056 participants, affecting all 12 items. Little’s MCAR test yielded a non-significant result (*χ*^2^ = 164.2; df = 166; *p* = 0.53), suggesting that the missing data were completely random. At the second measurement time point, there were 26 missing values (0.24%) across 16 (1.76%) of the 907 participants, affecting 9 out of 12 items. Little’s MCAR test yielded a non-significant result (*χ*^2^ = 91.7; df = 133 *p* = 1.00), supporting the hypothesis of missing completely at random data. At the third measurement time point, there were 18 missing values (0.17%) across 12 (1.39%) of the 866 participants, affecting 9 out of 12 items. Little’s MCAR test showed a non-significant result (*χ*^2^ = 87.319; df = 75; *p* = 0.16), supporting the MCAR assumption. At the fourth measurement time point, there were 32 missing values (0.31%) across the 21 (2.46%) of the 853 participants, affecting 10 out of the 12 items. Little’s MCAR test was non-significant (*χ*^2^ = 118.2; df = 132; *p* = 0.80), also supporting the MCAR hypothesis. The dropout rate did not significantly differ between digIRENA group (16.3%), IRENA group (22.8%), and the control group (19.1%) (*χ*^2^ = 5.1; df = 2; *p* = 0.08).

The demographic profile of the sample showed that approximately two-thirds (67.3%) were male, with the control group having the highest proportion at 73.6% (see Table 1). The mean age of the participants was 54.2 years, with the oldest participants in the control group (*M* = 55.7 years) and the youngest in the digIRENA group (*M* = 53.0 years). A vast majority of participants were German nationals (97.6%). More than three quarters (77.7%) were employed full-time. At the last measurement time point (t3), the participants in the digIRENA group (*M* = 1836 min; SD = 548 min) participated significantly more in the aftercare (*t* = 5.1; df = 317.6; *p* < 0.01) than the participants in the IRENA group (*M* = 1,492 min; SD = 826 min). A one-way ANOVA revealed no significant differences in the duration of structured physical activities outside of rehabilitation aftercare among the groups (*F* = 0.2; df_1_ = 2; df_2_ = 834; *p* = 0.78). Specifically, the mean duration for the digIRENA group was 80.6 min per week (SD = 74.3), for the IRENA group it was 85.3 min per week (SD = 120.4), and for the control group it was 85.4 min per week (SD = 87.9). There were also no significant differences between the digIRENA group (*M* = 11.84; SD = 3.88) and the IRENA group (*M* = 12.39; SD = 3.97) when comparing motivation in terms of the relative autonomy index before the start of the aftercare (*t* = 1.90; df = 746; *p* = 0.06).

**Table 1:** Sample characteristics at T0.

	digIRENA	IRENA	Control	Overall
Overall	405 (100%)	352 (100%)	299 (100%)	1,056 (100%)
Gender				
Male	260 (64.2%)	231 (65.6%)	220 (73.6%)	*711* (67.3%)
Female	145 (35.8%)	120 (34.1%)	79 (26.4%)	*344* (32.6%)
Divers	0	1 (0.3%)	0	1 (0.1%)
Missing	0	0	0	*0*
Age				
Mean	53.00	54.21	55.74	*54.18*
Standard deviation	8.75	7.90	6.30	*7.91*
*n*	405	351	299	*1,055*
Missing	0	1	0	*1*
Nationality				
German	398 (98.3%)	339 (96.3%)	294 (98.3%)	*1,031* (97.6%)
Non-German	7 (1.7%)	11 (3.1%)	4 (1.3%)	*22* (2.1%)
Missing	0	2 (0.6%)	1 (0.3%)	*3* (0.3%)
Employment status				
Full-time (>34 h)	318 (78.5%)	265 (75.3%)	238 (79.6%)	*821* (77.7%)
Part-time (15–34 h)	47 (11.6%)	43 (12.2%)	26 (8.7%)	*116* (11.0%)
Part-time (<15 h)	6 (1.5%)	2 (0.6%)	3 (1.0%)	*11* (1.0%)
Maternality/leave	0	0	0	*0*
Education	2 (0.5%)	2 (0.6%)	0	*4* (0.4%)
Unemployed	32 (7.9%)	37 (10.5%)	32 (10.7%)	*101* (9.6%)
Missing	0	3 (0.9%)	0	*3* (0.3%)
Participation in aftercare				
Mean	1836.24	1491.92		
Standard deviation	545.84	825.88		
*n*	263	196		
Missing	76	76		
Participation in other structured physical activities				
Mean	80.8	85.3	85.4	*83.5*
Standard deviation	74.3	120.4	87.9	*95.0*
*n*	331	270	236	*837*
Missing	74	82	63	
Motivation				
Mean	11.84	12.39		
Standard deviation	3.88	3.97		
*n*	401	347		
Missing	4	5		

### 3.2 Physical health

**Figure 1:**
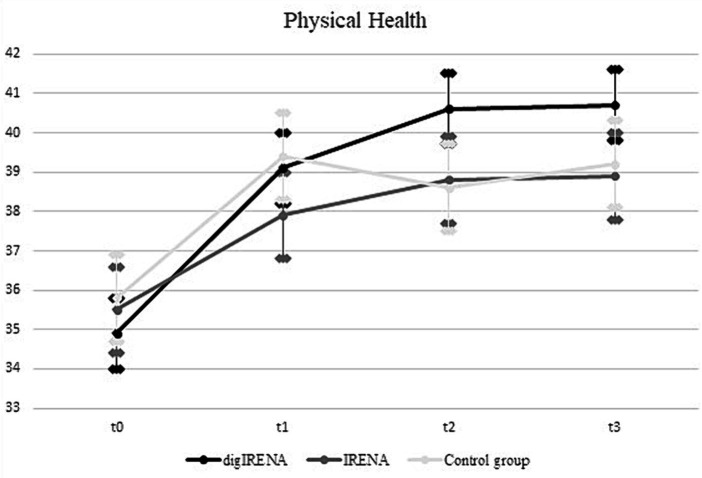
Development of physical health from T0 to T3. All error bars represent 95% CI.

The analysis incorporated data from a total of 805 participants who completed the SF-12 questionnaire at all time points. This included 323 participants from the digIRENA group, 252 from the IRENA group, and 230 from the control group (see Table 2). Figure 1 illustrates that the baseline mean scores were similar across the three groups, showing no significant differences (*F* = 2.7; df_1_ = 2; df_2_ = 1,052; *p* = 0.07). A significant main effect of time was observed (see Table 3), accounting for approximately 7.5% of the within-subject variance (*F* = 65.4; df_1_ = 2.4; df_2_ = 1955.0; *p* < 0.01; *η*^2^ = 0.075). Both the digIRENA and IRENA groups demonstrated a continuous increase in mean scores from T0 to T2, followed by a plateau (see Figure 1). In contrast, the control group exhibited an increase in mean scores from T0 to T1, after which a marginal decline was observed. A significant interaction effect for time * group was noted (*F* = 2.5; df_1_ = 4.9; df_2_ = 1955.0; *p* = 0.03; *η*^2^ = 0.006), with the digIRENA group displaying a steeper incline in mean scores from T0 to T2 compared to the other two groups (see Figure 1). However, the effect size was rather small, explaining only 0.6% of the variance in physical health.

**Table 2:** Descriptive statistics for physical health.

	T0					T1			
Group	*n*	*M*	SD	LL	UL	*M*	SD	LL	UL
digIRENA	323	34.9	8.9	33.9	35.9	39.1	9.3	38.1	40.1
IRENA	252	34.5	8.2	33.5	35.5	37.9	9.4	36.8	39.1
Control	230	35.8	8.2	34.7	36.9	39.4	9.1	38.2	40.6
	T2					T3			
digIRENA	323	40.6	9.7	39.6	41.7	40.7	10.1	39.6	41.8
IRENA	252	38.8	9.9	37.6	40	38.9	9.7	37.7	40.1
Control	230	38.6	9.3	37.4	39.8	39.2	10.2	37.9	40.5

*n*, number of subjects; *M*, Mean; SD, standard deviation.

**Table 3:** Analysis of variance for physical health T0–T3.

	SS	*df*	MSS	*F*	*p*	*η*^2^
Time	10540.7	2.4	4324.1	65.4	0.00	0.075
Time * Group	812.6	4.9	166.7	2.5	0.03	0.006
Error	129321.5	1955.0	66.1			

SS, Sum of Squares; df, Degrees of Freedom; MSS, Mean Sum of Squares; F, *F*-value; *η*^2^, Effect Size.

**Figure 2:**
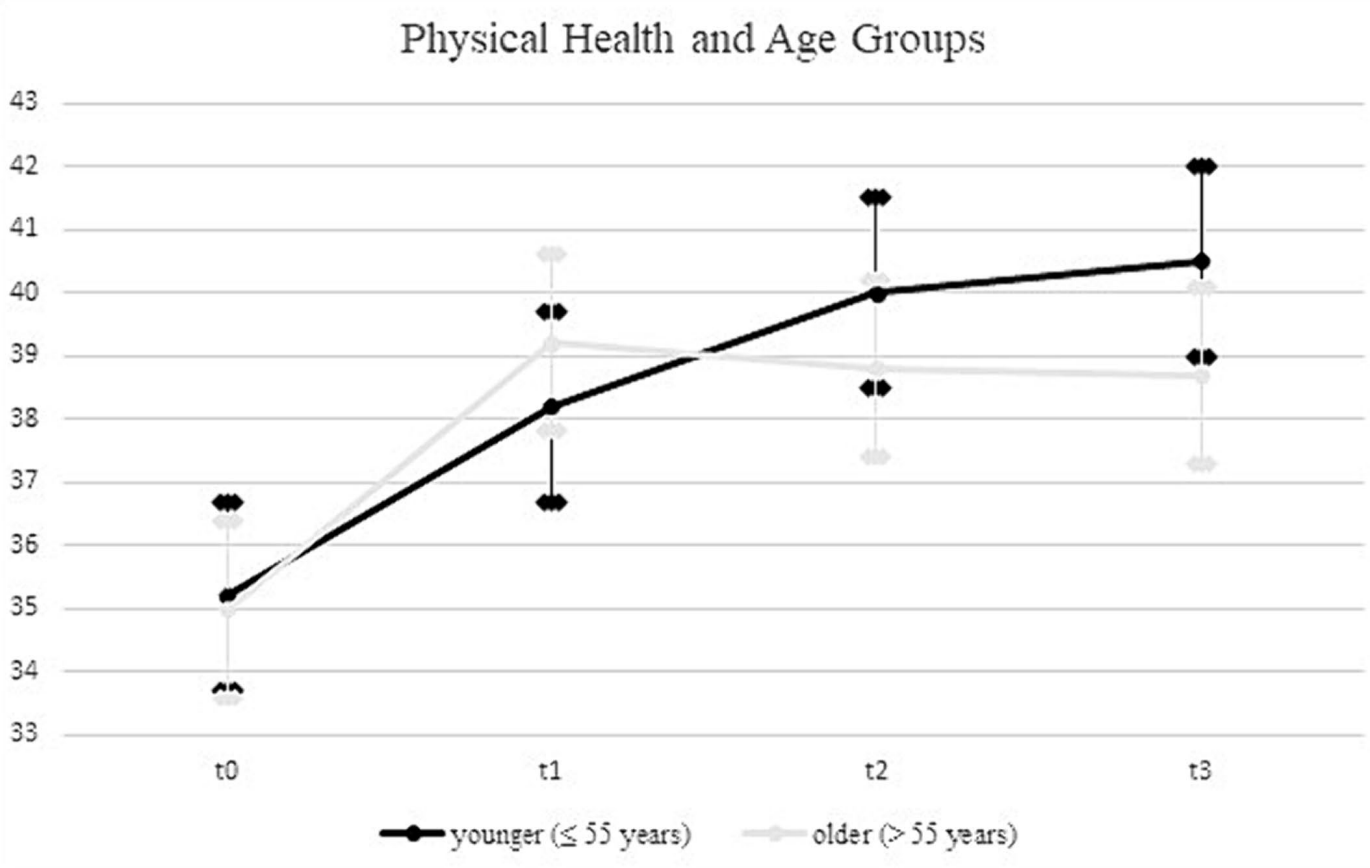
Development of physical health for age groups. All error bars represent 95% CI.

Upon incorporating age as a covariate and gender as an additional factor in the ANOVA model (see Table 4), the previously significant time * group interaction was no longer statistically significant (*F* = 1.3; df_1_ = 3.7; df_2_ = 1526.3; *p* = 0.29; *η*^2^ = 0.003). In contrast, a significant time * age interaction emerged, explaining 0.9% of the variance in physical health (*F* = 7.6; df_1_ = 1.8; df_2_ = 1526.3; *p* = 0.00; *η*^2^ = 0.009). Using the median split for the age variable, the follow-up analyses showed a more continuous increase for the younger rehabilitants (≤55 years) across all four measurement time points, while for the older rehabilitants (>55 years) there was a decrease in values from the second measurement time point (see Figure 2). In a separate ANOVA, we probed the three-way interaction effect of time * age * group, which yielded no significant results (*F* = 0.4; df_1_ = 4.9; df_2_ = 1936.4; *p* = 0.87; *η*^2^ = 0.001).

**Table 4:** Analysis of variance for physical health including age and gender.

	SS	df	MSS	*F*	*p*	*η* ^2^
Time	2184.6	2.4	892.3	13.7	0.00	0.017
Time * Age	1464.4	2.4	598.1	9.2	0.00	0.011
Time * Group	344.8	4.9	70.4	1.1	0.37	0.003
Time * Gender	188.8	2.4	77.1	1.2	0.31	0.001
Time * Group * Gender	148.8	4.9	30.4	0.5	0.80	0.001
Error	127460.1	1951.3	65.3			

SS, Sum of Squares; df, Degrees of Freedom; MSS, Mean Sum of Squares; F, *F*-value; *η*^2^, Effect Size.

### 3.3 Mental health

**Figure 3:**
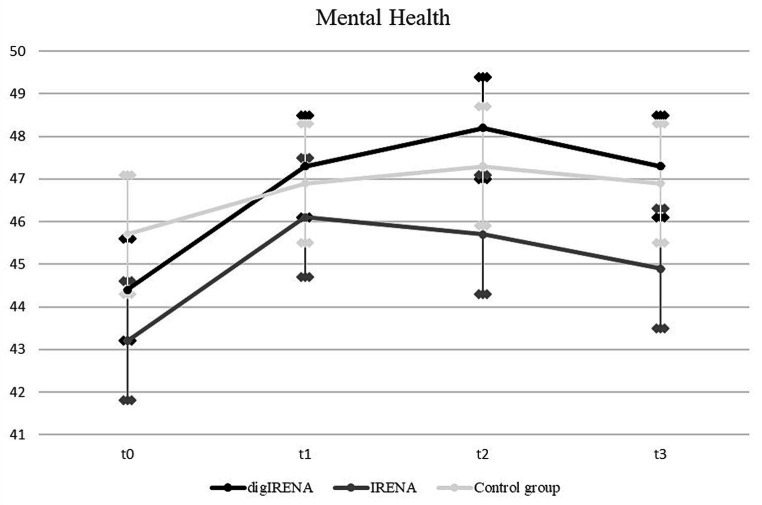
Development of mental health from T0 to T3. All error bars represent 95% CI.

Data were analyzed for a total of 805 participants who completed the SF-12 questionnaire at all measurement occasions. This included 323 participants from the digIRENA group, 252 from the IRENA group, and 230 from the control group (see Table 5). The results indicated that at baseline (T0), participants in the control group had significantly higher scores compared to both the digIRENA and IRENA groups, with digIRENA participants scoring higher than those in the IRENA group (*F* = 4.0; df_1_ = 2; df_2_ = 1,052; *p* = 0.02). All three groups exhibited an increase from T0 to T1, reflected in a significant main effect of time that accounted for approximately 1.8% of within-subjects variance (see Table 6). Participants in the digIRENA group demonstrated the steepest rise from T0 to T2 (see Figure 3). However, a decline in scores was observed for all groups beyond T2. Overall, the time * group interaction was not statistically significant (*F* = 1,1; df_1_ = 4.7; df_2_ = 1890.7; *p* = 0.34; *η*^2^ = 0.003).

**Table 5:** Descriptive statistics for mental health.

	T0					T1			
Group	*n*	*M*	SD	LL	UL	*M*	SD	LL	UL
digIRENA	323	44.4	11.2	43.2	45.6	47.3	10.9	46.1	48.5
IRENA	252	43.2	11.4	41.8	44.6	46.1	11.0	44.7	47.4
Control	230	45.7	10.4	44.4	47.0	46.9	10.8	45.5	48.3
	T2					T3			
digIRENA	323	48.2	10.7	47.0	49.4	47.3	11.2	46.1	48.5
IRENA	252	45.7	11.7	44.2	47.1	44.9	11.9	43.4	46.4
Control	230	47.3	10.8	45.9	48.7	46.9	10.7	45.5	48.3

*n*, Number of Participants; *M*, Mean; SD, Standard Deviation; LL, Lower Limit; UL, Upper Limit.

**Table 6:** Analysis of variance for mental health T0–T3.

	SS	*df*	MSS	*F*	*p*	*η* ^2^
Time	3284.7	2.4	1393.3	14.3	0.00	0.018
Time * Group	519.6	4.7	110.2	1.1	0.34	0.003
Error	183634.0	1890.7	97.1			

SS, Sum of Squares; df, Degrees of Freedom; MSS, Mean Sum of Squares; F, *F*-value; *η*^2^, Effect Size.

When age was included as a covariate and gender as an additional factor in the ANOVA, the main effect of time was not significant (*F* = 1.0; df_1_ = 2.3; df_2_ = 1874.2; *p* = 0.39; see Table 7). Notably, only the interaction of gender * group exhibited a significant effect (*F* = 4.6; df_1_ = 2.4; df_2_ = 1874.2; *p* = 0.01; *η*^2^ = 0.006). Subsequent analyses revealed that this effect was attributable to female participants having significantly lower baseline scores compared to males. During the follow-up period, the mean scores for males and females converged. In a separate ANOVA, it was determined that the three-way interaction of time * group * age had no significant effect on mental health outcomes (*F* = 1.9; df_1_ = 4.7; df_2_ = 1871.1; *p* = 0.09; *η*^2^ = 0.006).

**Table 7:** Analysis of variance for mental health T0-T3 including age and gender.

	SS	*df*	MSS	*F*	*p*	*η* ^2^
Time	221.8	2.3	94.3	1.0	0.39	0.001
Time * Age	38.0	2.3	16.2	0.2	0.88	0.000
Time * Group	367.4	4.7	78.1	0.8	0.54	0.002
Time* Gender	1040.8	2.4	442.6	4.6	0.01	0.006
Time * Group * Gender	319.8	4.7	68.0	0.7	0.62	0.002
Error	182138.5	1874.2	97.1			

SS, Sum of Squares; df, Degrees of Freedom; MSS, Mean Sum of Squares; F, *F*-value; *η*^2^, Effect Size.

## 4 Discussion

The main aim of the present study was to evaluate the efficacy of a digital aftercare program in improving mental and physical health outcomes. Therefore, the study examined three groups: a digital aftercare group (digIRENA), a traditional rehabilitation aftercare group (IRENA), and a control group without an aftercare intervention. We aimed to determine not only the relative effectiveness of digIRENA in improving health indicators, but also how it compares to traditional aftercare approaches and the absence of aftercare.

A notable observation from our study was the improvement in physical health measures for both the IRENA and digIRENA groups, with a statistically significant increase in SF-12 physical health subscale scores contributing to approximately 7.5% of intraindividual variance. While the rate of improvement appeared slightly more pronounced in the digIRENA group, this finding warrants careful interpretation in light of the overall similarities between the groups and the observed decline in SF-12 scores after T1. Given the unique circumstances of the COVID-19 pandemic, which may have influenced participants’ willingness to engage in traditional face-to-face programs, it is prudent to consider these factors when evaluating the relative effectiveness of digitized aftercare approaches. This context highlights the importance of digital health interventions as a complement to traditional care, especially in scenarios where conventional approaches may be less accessible.

While our findings indicate significant improvements in physical health measures for both the IRENA and digIRENA groups, it is crucial to contextualize these improvements in light of similar progress observed in the control group between T0 and T1. The control groups’ enhancement, demonstrating a parallel pattern of improvement, raises important considerations regarding the specific contribution of the intervention programs. This observation compels us to carefully evaluate the extent to which the improvements in the intervention groups can be attributed solely to the program’s effects, as opposed to natural recovery processes over time or general health trends among the study population. The presence of improvement in the control group suggests that factors beyond the structured aftercare contributed to health gains, which may include natural recovery, seasonal effects, or changes in individual health behaviors independent of our study interventions. Additionally, the participation of control group members in structured physical activities outside of the aftercare programs may also have contributed to the observed improvements in their health outcomes.

In terms of mental health, participants in the control group displayed higher baseline mental health scores compared to their counterparts in the digIRENA and IRENA groups. Such disparities at baseline, likely due to self-selection, could be explained by the randomization process and are similar to problems documented in other intervention studies (40). As reflected in the lower number of individuals that were initially in the control group compared to the digIRENA-group, there might have been an attrition bias. Although due to logistic reasons we did not keep track of these patterns, it might be that individuals who withdrew their willingness to participate in the study after they had been informed that they were in the control group might have had lower mental health scores than those who decided to stay in the study. While all groups demonstrated a marked improvement from T0 to T1, the digIRENA group showed the greatest increase at T2, mirroring the findings of Gold et al. (41) that underscore the accelerated benefits of digital interventions. Nevertheless, the absence of a significant interaction effect between time and group mitigates these observations.

In addition, one of the surprising findings of our study was the decline in mental health outcomes observed in all groups at 4 months after the end of the aftercare intervention. Although the digIRENA and IRENA groups maintained scores above their initial baseline, the general decline postulates a transient nature of the positive effects conferred by the rehabilitation. This trend aligns with findings of Winzer et al. (42), who identified a diminishing effect of mental health interventions over time if not bolstered by ongoing support or periodic follow-up sessions. Similarly, Bond and colleagues (43) observed that the benefits of mental health interventions, especially in the context of rehabilitation, can be short-lived, emphasizing the significance of continued care and strategies to promote long-term mental well-being. Our results, in light of these previous studies, advocate for the integration of sustained, perhaps even aftercare concepts with refresher workshops at regular intervals to ensure enduring positive outcomes in mental health.

With the inclusion of age and gender into the analysis, the main effect of time disappeared. This observation parallels findings by Schmidt et al. (39), underscoring the importance of demographic variables in modulating responses to digital interventions. Notably, an interaction effect between time and gender was discerned. Women, who had lower mental health scores than men at T0, manifested the most pronounced improvements throughout the rehabilitation period. This trend is consistent with the growing body of evidence highlighting gender-specific trajectories in response to mental health interventions (44). Consequently, these insights emphasize the need to integrate gender-specific considerations when delivering rehabilitation programs.

In our analysis of mental health outcomes, it is crucial to consider the unique backdrop against which this study was conducted—the COVID-19 pandemic (45). The absence of significant differences in mental health improvements across groups, despite the varied intervention modalities, may partly reflect the pervasive stress and uncertainty induced by the pandemic. This period has been marked by widespread reports of increased psychological distress across all demographics (46), which could obscure the mental health benefits typically conferred by aftercare programs. The pandemic’s impact could especially affect older adults, who not only were advised to avoid social contacts but also might have experienced heightened concerns for their health, further influencing their mental well-being and responsiveness to rehabilitation efforts.

For physical health, an interaction effect between time and age was revealed, indicating a heterogeneous response to the rehabilitation programs depending on age. Specifically, younger participants exhibited a sustained trajectory of improvement across all measurement occasions, whereas their older counterparts reached a plateau following an initial upswing. This finding that younger patients appear to benefit more from interventions aligns partially with findings in the broader rehabilitation literature. While Gosselin et al. (47) found that intensive rehabilitation programs were equally beneficial for young and older participants, the study by Palmcrantz et al. (48) found that younger individuals tended to receive more care and rehabilitation services, suggesting a possible inequity in the allocation of health resources. Nonetheless, it was observed that self-perceived global recovery did not significantly vary between different age groups.

The present study found that the three-way interaction of time, group, and age was not significant for both physical and mental health outcomes. This finding differs from that of Schmidt et al. (39), who reported that younger individuals derived greater benefits from digital prevention compared to their older counterparts. The absence of a significant interaction in our data suggests that the effectiveness of the digIRENA program may not be moderated by age, thus challenging the assumption that digital interventions are particularly advantageous for younger cohorts. This discrepancy emphasizes the complexity of age as a variable in the context of healthcare interventions and underlines the need for more nuanced investigations into the demographic-specific effects of digital rehabilitation programs.

An important consideration in our study’s context is the overarching impact of the COVID-19 pandemic on both the implementation of aftercare programs and the mental health of participants. Our findings indicated that participants in the digIRENA group engaged significantly more in the aftercare program compared to those in the IRENA group. Since a significant body of research suggests that mental health can affect adherence (49, 50), one might hypothesize that this increased engagement could be due to better mental health among digIRENA participants. However, our analysis revealed that the mental health scores at the onset of aftercare did not significantly differ between the digIRENA and IRENA groups, suggesting other factors influenced participants’ engagement levels.

The flexibility offered by digIRENA, allowing for immediate commencement post-rehabilitation clinic stay, appears to be a critical factor in this increased participation. This advantage became particularly relevant during the COVID-19 pandemic when concerns about face-to-face interactions may have dissuaded participants from engaging with traditional IRENA sessions. Such apprehensions, along with logistical challenges presented by pandemic-related restrictions, underscore the importance of digital aftercare solutions like digIRENA, which bypass many of the barriers inherent to in-person treatment modalities. Moreover, the pandemic’s effect on mental and physical health—a significant concern during this period—raises questions about the interplay between participants’ health status, their motivation for aftercare participation, and the overall effectiveness of the interventions. The control group’s higher baseline mental health scores compared to the intervention groups, and particularly the IRENA group’s lower scores, highlight the importance of mental health in influencing treatment compliance and engagement.

Previous evaluations of the IRENA program, notably by Lamprecht et al. (15, 51), before the emergence of the COVID-19 pandemic, offer insights into its effectiveness in improving participants’ health status. The 2011 study indicated that 79% of participants reported an improvement in their health status following IRENA, with significant enhancements in physical and mental health domains. However, these outcomes were based on retrospective data, with participants evaluating their condition improvement post-IRENA without a control group for comparison. The 2012 study, which also lacked a control group, did not observe significant changes in health-related quality of life using the SF-12 1 year post-IRENA, suggesting the potential for transient effects of the intervention. Comparing these previous findings to our current study, which includes a control group and pre-post measurements, offers a nuanced understanding of IRENA’s effectiveness amid the pandemic. Our results suggest that while digital aftercare and traditional IRENA can both lead to improvements in physical and mental health outcomes, the context of COVID-19 has introduced unique challenges and considerations. Specifically, the increased engagement in digIRENA over IRENA may not solely be attributed to differences in health status but perhaps to the digital program’s flexibility and ability to circumvent pandemic-related barriers to participation.

### 4.1 Strengths and limitations

The present study has several strengths. First, the large sample size supports the robustness of our findings, increasing their generalizability and reducing the margin of error inherent in smaller samples. Second, the use of validated measures to assess health outcomes was a strength. Moreover, the longitudinal design allows to assess the development of the individual indicators across time. Third, our study design included two intervention groups (digIRENA and IRENA) and a control group, which allowed us to estimate the effectiveness of digital aftercare. These methodological strengths allow for a comprehensive comparison of the digIRENA, IRENA, and control group.

However, several limitations need to be taken into account. Primarily, the adoption of partial randomization, necessitated by ethical considerations, introduces the possibility of self-selection bias. This bias may manifest as participants with limited digital proficiency opting for traditional IRENA. Secondly, as previously mentioned, the observed discrepancy in initial participant numbers between the digIRENA and control groups may indicate an attrition bias related to group allocation. These factors collectively highlight the challenges in achieving a perfectly balanced experimental design and necessitate a cautious interpretation of the findings within these constraints. Thirdly, the study design may not have captured the true intervention period, given the variability in the commencement of aftercare measures. Additionally, our exclusive reliance on self-report measures might have introduced biases. Incorporating objective metrics, such as actual days of work incapacity, could enrich future studies. Furthermore, we did not systematically collect data on the specific reasons participants declined participation in the traditional IRENA program. This oversight limits our ability to fully understand the range of factors influencing individuals’ decisions against engaging with this aftercare option. Moreover, the unprecedented conditions of the COVID-19 pandemic introduced significant extraneous variables that may have impacted the study’s outcomes. The pandemic’s influence extended beyond the mere availability of traditional IRENA courses, potentially affecting participants’ willingness to engage in face-to-face interventions due to health concerns. This could have led to a preferential decline in IRENA participation or suboptimal engagement during sessions, motivated by fears of contracting the virus. Moreover, the pandemic’s pervasive impact on public health measures and personal well-being might have influenced both mental and physical health outcomes, introducing motivational deficits that hinder proper participation in the aftercare programs (52). In addition, a notable limitation of our study is that the control group independently engaged in structured physical activities, effectively creating their own intervention. This unforeseen variable may have diminished the apparent effects of the structured IRENA and digIRENA programs, as the control group’s self-directed activities contributed to health improvements that could mask the distinct benefits of the formal interventions. Finally, this study did not account for the varying contextual conditions of the participants, such as their living environments, availability of informal care, and social support systems. These factors could significantly influence rehabilitation outcomes by affecting patients’ ability to engage with aftercare programs and adhere to recommended practices.

### 4.2 Implications and future directions

The findings from this study have several implications for health rehabilitation research and its applied domains. One of the observations concerns the effectiveness of the digIRENA program, which highlights the potential of digital interventions in the rehabilitation sector (22). In today’s digital age, healthcare professionals have the opportunity to seamlessly integrate traditional rehabilitation methods with digital alternatives, allowing them to expand and improve aftercare services (27). This is particularly relevant when addressing the rehabilitation needs of younger cohorts, who are often more adept and receptive to technological interfaces (53). Moreover, the nuances in rehabilitation responses, as reflected in age and gender differences, underscore the urgency of individualized intervention designs. Relying on generalized, standardized approaches may inadvertently attenuate the efficacy of interventions failing to take into account the heterogeneity of rehabilitation recipients (54). Furthermore, it becomes imperative to delve deeper into understanding how digital aftercare influences different forms of motivation, including intrinsic and extrinsic motivation. Such exploration could unveil critical insights into how motivational factors contribute to the adherence and overall success of rehabilitation programs, offering guidance for the design of more effective, engaging, and personalized aftercare strategies.

Despite the observed benefits of the digIRENA program in our study, it is important to recognize the inherent limitations of telerehabilitation, which have been highlighted by existing research. Challenges such as technological barriers, the necessity for patient and provider digital literacy, potential reductions in the therapeutic relationship due to the absence of in-person interactions, and concerns regarding data security and privacy remain significant (19, 20). Moreover, the impersonal nature of digital interventions may not suit all individuals, especially those who derive substantial therapeutic benefit from direct human contact. While our findings suggest a role for telerehabilitation in expanding access to aftercare services, especially under constraints such as those imposed by the COVID-19 pandemic, these limitations underscore the need for a nuanced approach to integrating digital solutions into the rehabilitation continuum. It is essential to balance the benefits of digital aftercare with the value of traditional, face-to-face interactions, ensuring that rehabilitation programs are accessible, effective, and respectful of patient preferences and needs (55).

## 5 Conclusion

The landscape of health rehabilitation is in a state of dynamic change, driven by the growing possibilities of digital interventions. The present study sought to compare the effectiveness of a novel digital aftercare program (digIRENA) with its traditional counterpart (IRENA) and a control group. The results highlight not only the potential of digital modalities to improve rehabilitation aftercare outcomes, but also the intricate interplay of demographic variables such as age and gender in shaping these outcomes. The study demonstrates that digital interventions like digIRENA can be effective and serve as valuable complements to conventional aftercare methods. In addition, the observed temporary improvement in mental health outcomes highlights the need for interventions that provide ongoing support. By shedding light on these aspects, this research contributes to digital health research and invites further investigations in this promising field.

## Data availability statement

The raw data supporting the conclusions of this article will be made available by the authors, without undue reservation.

## Ethics statement

The studies involving humans were approved by Ethikkommission des Karlsruher Instituts für Technologie. The studies were conducted in accordance with the local legislation and institutional requirements. The participants provided their written informed consent to participate in this study.

## Author contributions

DS: Conceptualization, Formal analysis, Funding acquisition, Investigation, Resources, Software, Visualization, Writing – original draft, Writing – review & editing. JF: Data curation, Investigation, Project administration, Writing – review & editing. KF: Writing – review & editing. SW: Writing – review & editing. L-MR: Writing – review & editing. DJ: Conceptualization, Funding acquisition, Methodology, Resources, Supervision, Writing – review & editing.

## Footnotes

^1^ https://caspar-health.com/en-us
